# Supplementary material for: Exosomal MicroRNAs as Epigenetic Biomarkers for Endometriosis: A Systematic Review and Bioinformatics Analysis
Source: Int J Mol Sci. 2025 May 9;26(10):4564. doi: 10.3390/ijms26104564 (PMC12111455; doi:10.3390/ijms26104564)
Supplement: Supplementary file 1 [file ijms-26-04564-s001.zip › Table S2 Search strategy.pdf]

Table S2: Search strategy.

PubMed	Total Number of Papers
(((("extracellular vesicles"[MeSH Terms] OR ("extracellular"[All Fields] AND "vesicles"[All Fields]) OR "extracellular vesicles"[All Fields] OR ("exosomal"[All Fields] OR "exosomes"[MeSH Terms] OR "exosomes"[All Fields] OR "exosome"[All Fields] OR "exosomic"[All Fields]) OR ("exosomal"[All Fields] OR "exosomes"[MeSH Terms] OR "exosomes"[All Fields] OR "exosome"[All Fields] OR "exosomic"[All Fields]) OR ("extracellular vesicles"[MeSH Terms] OR ("extracellular"[All Fields] AND "vesicles"[All Fields]) OR "extracellular vesicles"[All Fields] OR ("extracellular"[All Fields] AND "vesicle"[All Fields]) OR "extracellular vesicle"[All Fields]))	
(("endometriosis"[MeSH Terms] OR "endometriosis"[All Fields] OR "endometrioses"[All Fields])	
(("microrna s"[All Fields] OR "micrornas"[MeSH Terms] OR "micrornas"[All Fields] OR "microrna"[All Fields])) OR ("micrornas"[MeSH Terms] OR "micrornas"[All Fields] OR "mirna"[All Fields] OR "mirnas"[All Fields] OR "mirna s"[All Fields])	
#1 AND #2 AND #3	477
**Web of Sciences**	
((((ALL=(microRNA)) OR ALL=(micro RNA)) OR ALL=(microRNAs)) OR ALL=(miRNA)) OR ALL=(miRNAs)	
((ALL=(endometriosis)) OR ALL=(adenomyosis externa)) OR ALL=(endometriosis externa)	
(((ALL=(exosome)) OR ALL=(exosomes)) OR ALL=(extracellular vesicle)) OR ALL=(extracellular vesicles)	
#1 AND #2 AND #3	49
**SCOPUS**	
TITLE-ABS-KEY ("microRNA" OR "micro RNA" OR "microRNAs" OR "miRNA" OR "miRNAs")	
TITLE-ABS-KEY ("endometriosis" OR "adenomyosis externa" OR "endometriosis externa")	
TITLE-ABS-KEY ("exosome" OR "exosomes" OR "extracellular vesicle" OR "extracellular vesicles")	
#1 AND #2 AND #3	88

Embase	
'microRNA' OR 'micro RNA' OR 'microRNAs' OR 'miRNA' OR 'miRNAs'	
'endometriosis' OR 'adenomyosis externa' OR 'endometriosis externa'	
'exosome' OR 'exosomes' OR 'extracellular vesicle' OR 'extracellular vesicles'	
#1 AND #2 AND #3	88
